# Incorporation of the Multi-Layer Plastic Packaging in the Asphalt Binders: Physical, Thermal, Rheological, and Storage Properties Evaluation

**DOI:** 10.3390/polym14245396

**Published:** 2022-12-09

**Authors:** Ali Qabur, Hassan Baaj, Mohab El-Hakim

**Affiliations:** 1 Department of Civil and Environmental Engineering, University of Waterloo, Waterloo, ON N2L 3G1, Canada; 2 Department of Civil and Environmental Engineering, Manhattan College, Bronx, NY 10471, USA

**Keywords:** multi-layer plastic packaging (MPP), recycling, asphalt cement, fatigue, permanent deformation, circular economy

## Abstract

The amount of residual Multi-layer Plastic Packaging (MPP) in Canada has greatly increased in the last two decades, which has economic and environmental consequences. MPP is primarily made up of two or more layers of Polyethylene (PE), Polyester (PET), Nylon (NY), and Metalized Polyester (METPET). While MPP has not been used as an asphalt modifier, some of the materials commonly found in MPP, such as PE and PET, have also been successfully used as asphalt modifiers. Nevertheless, a few recent studies have demonstrated the potential for reusing MPP as an asphalt modifier to improve asphalt pavement performance. Recycling post-industrial MPP instead of using raw polymers could lead to economic and environmental benefits. However, a comprehensive study to evaluate MPP as a viable asphalt additive is lacking. The main objective of this study is to evaluate the feasibility of using MPP as an asphalt modifier via the wet method, considering the physical, thermal, rheological, and storage properties of the MPP-modified binder at different MPP concentrations (2%, 4%, and 8%) in asphalt cement (PG 58–28). MPP-modified binders were evaluated using the following instruments: Differential Scanning Calorimeter (DSC), Thermogravimetric Analysis (TGA), Superpave Dynamic Shear Rheometer (DSR), Rotational Viscosity (RV), and Environmental Scanning Electron Microscopy (ESEM). Test results indicated that the incorporation of MPP has a strong potential to improve permanent deformation resistance at high temperatures. In addition, MPP shows a moderate impact on fatigue cracking performance at intermediate temperatures. Overall, in low-temperature climates, using less than 4% of MPP additives would offer higher fatigue damage resistance along with adequate permanent deformation. In high-temperature climates, higher concentrations of additives may be preferable to resist permanent deformation. Finally, MPP is a challenge for existing recycling systems, and its incorporation into asphalt applications may develop more sustainable materials that would contribute to circular economy principles.

## 1. Introduction

Canada produces 3.3 million tons of waste plastic per year, of which approximately 2.8 million tons end up in Canadian landfills every year [1]. The disposed plastic waste represents about 86% of all leftover plastic which mainly contains polypropylene (PP), polyethylene terephthalate ‘Polyester’ (PET), polyethylene (PE), low-density polyethylene (LDPE), and high-density polyethylene (HDPE) [1]. The disposal of this waste plastic in landfills represented a lost opportunity of approximately CAD $7.8 billion in 2016 alone, and it will rise to CAD $11.1 billion by 2030 [1]. The development of a reliable method to reduce unrecovered plastic is essential. Plastic recycling should be performed in a cost-saving manner that reduces the carbon footprint of plastic production.

Elastomer/Plastomer or thermoplastic types of polymer are successfully used in asphalt modification [2]. Former research investigations reported that thermoplastic-based polymers are suitable for producing polymer-modified asphalt. In the last two decades, there has been a significant amount of research investigating the single-use plastic type of waste plastic polymers in asphalt modification such as LDPE, HDPE, PP, PE, and PET. Virgin polymers are known to improve asphalt binder properties. However, they are used in small modification percentages for financial constraints and due to a lack of knowledge of their impact on the mechanistic and rheological properties of the binder [3]. Using waste polymer as an asphalt modifier instead of virgin polymers has shown similar results in terms of improving mixture performance with reduced environmental and financial disadvantages. During the past four decades, studies focused on the use of waste plastic polymers in asphalt. Polypropylene (PP) was investigated by several scholars as a recycled additive in asphalt mixtures [4,5,6]. Polyethylene (PE) can be found in several applications, including low- and high-density polyethylene which is used in plastic bottles, packaging, and single-use plastic bags [7]. According to Hinisliglu and Agar, when the waste plastic of HDPE was used as a polymer modifier, using various mixing times (5 min, 15 min, and 30 min), temperatures (145 °C, 155 °C, and 165 °C), and percentages of HDPE (4%, 6%, and 8% of the weight of asphalt binder), the binders had higher stability, strength, and a higher Marshall quotient value which improved the resistance to permanent deformation.

In addition, the optimum result for Marshall stability was achieved at 4% HDPE binder modification by weight and mixed for 30 min at 165 °C [8]. Garcia-Morales et al. [9] found a similar result to that of Hinsisliglu and Agar’s study of the rheology of recycled polymer-modified asphalt cement. The Garcia-Morales et al. study used flow behavior of 60/70 penetration grade asphalt binder modified with 5% and 9% waste EVA/LDPE at high temperatures and linear viscoelasticity, and low and intermediate temperatures. The study showed the modified recycled EVA/LDPE asphalt binder improved the mechanical properties and overall performance of road service life. According to Casey et al. [10], the addition of 4% waste HDPE resulted in achieving the optimum performance in fatigue and rutting resistance. Hadidy and Yi-qiu [11] confirmed the findings of the aforementioned studies, namely, that the improvement of the softening point was directly related to an improvement in permanent deformation. Furthermore, the ductility result from the addition of LDPE in the modified asphalt binder showed an improvement in the cement performance in both high and low-temperature regions. According to Fang, binder modification using both polyethylene packaging waste (WPE) and organophilic montmorillonite (OMMT) significantly improved the fundamental properties of the modified asphalt, including the high-temperature performance, and low-temperature cracking resistance [12]. Maharaj concluded that the particle size and the amount of polyethylene terephthalate (PET) used had an impact on the asphalt binder in terms of fatigue cracking resistance and rutting resistance [13]. Xu et al. conducted a study on waste PET which is chemically treated by using triethylenetetramine (TETA) and ethanolamine (EA). This study also confirmed the capability of incorporating chemically treated waste PET into rubberized bitumen. The findings showed that waste PET improved the overall performance of rubberized bitumen [14].

Wang’s study on the use of polyethylene (PE) and crumb tire rubber (CTR) found that the rheological properties of the asphalt binder were enhanced after modification at different temperature ranges. To acquire a stable modified asphalt binder, the density difference must be reduced, and the interaction must be enhanced [15]. However, there was a slight difficulty in the compatibility between waste polyethylene packaging (WPE) and asphalt cement when WPE exceeded 10% [16]. Furthermore, there was no improvement in asphalt binder performance at low temperatures through binder modification using waste tire rubber (WTR) and reclaimed low-density polyethylene (RPE) [17]. Table 1 summarizes some studies that investigated the use of waste plastic additives via the wet method (into asphalt binders).

The overall finding of this research suggested that recycled plastic can be included as: (i) a substitute for aggregates, (ii) an aggregate coating, (iii) an asphalt binder modifier, or some combination of the three [18,19,20,21,22]. This incorporation of waste plastic additives in asphalt binder and/or asphalt mixture can improve the physical and mechanical properties of road pavements in terms of fatigue and rutting. The current study examined the use of MPP powder as a binder additive and its effect on the physical, thermal, rheological, and storage properties of the modified binder.

**Table 1:** Incorporation of Plastic Waste Additive to Asphalt Binder.

							Mixing Conditions				
Origin	Plastic Type	Density (g/cm^3^)	Tm ^1^ (°C)	Shape/Size (mm)	Binder Grade	OPT (%)	MT ^2^ (°C)	Mix Speed (RPM)	Time (min)	Notes	REF ^3^
Computer parts	Electronic- Acrylonitrile Butadiene Styrene (ABS), Acrylonitrile Butadiene Styrene-Polycarbonate (ABS-PC) and High Impact Polystyrene (HIPS)	N/A	ABS = 105, ABS-PC = 125, HIPS = 180–260	Powder/0.3	PG58–28	5	N/A	5000 + 3000	45 + 15	E-waste plastics were treated with cumene hydroperoxide. The results showed untreated e-waste modified asphalt binders were stiffer and had more elastic behavior than the control binder; however, in treated e-waste plastics, the increases were significantly higher. Thus, treated e-waste modifiers have significantly improved the resistance to rutting of asphalt binders than untreated.	[23]
Waste petrochemical	Recycle Waste Polyethylene (RPE)	RPE = 0.92	RPE = 190	Powder/N/A	Aryl Hydrocarbon Bitumen AH-70	4	180	2000 + 5000 + <100	20 + 90 + 30	After adding 2% of RPE into asphalt binders, the performance grade changed and enhanced at the high-temperature performance, whereas at the low-temperature, the performance was kept unchanged after modification.	[24]
Waste packaging	Waste Polyethylene (WPE)	N/A	WPE = N/A	Powder/N/A	Non-waxy crude only A90	4	150, 175, 190, 205	3700	90	190 °C was the most suitable and recommended preparation temperature to mix WPE into the asphalt binder.	[25]
Waste milk packaging	Waste Packaging Polyethylene (WPE)	WPE = 1.8	WPE = N/A	Powder/N/A	A90	4	150	3750	90 min (with 10-min rest periods every half hour)	Organic montmorillonite (OMt) was mix with WPE modified asphalt. The results revealed that the addition of OMt improved the storage stability of WPE-modified asphalt, and meanwhile, OMt does not compromise WPE-modified asphalt’s excellent high-temperature rheological properties.	[26]
Waste bottles	High-density polyethylene (HDPE)	N/A	HDPE = N/A	Powder/0.149–0.074	PG 64–16	10	180	4500	40	When 6 and 10% of HDPE were added to the asphalt binder, the fatigue life was improved.	[27]
Waste bottles	Waste rubber and polypropylene (PP), a blend of crumb rubber (CR) and PP powder by a ratio of 40:1 mixed with base asphalt to form plastic rubber asphalt (PRA)	PP = N/A, and CR = N/A	PP = N/A, and CR = N/A	PP and CR = Powder/Max 0.6 to 0.05	Shell 70	20	190	3600	N/A	Using plastic–rubber asphalt PRA mixture was matched with the SBS mixture for the low, high-temperature performances and water susceptibility, and it was more environmentally friendly in terms of energy consumption and greenhouse gas GHGs.	[28]
Waste pipe	Waste polyvinylchloride (PVC)	PVC = N/A	PVC = N/A	PVC = Powder/2–4	80/100	5	N/A	2000	120–180	The addition of waste PVC increased the rutting and fatigue life resistance of the asphalt mix.	[29]
Waste packaging	Waste polyethylene packaging (WPE)	WPE = N/A	WPE = N/A	WPE = Powder/4	N/A	6	N/A	3600	120	Modified asphalt with 10 wt% and below of WPE was the recommended percentage to obtain better service performances.	[16]
Waste packaging	Recycle polypropylene (PP), high- and low-density polyethylene (HDPE), and (LDPE)	PP, HDPE and LDPE = N/A	PP = 162, HDPE = 132 and LDPE = 110	PP, HDPE and LDPE = N/A	PG 64–22	4	PP = 190, HDPE = 180 and LDPE = 160	5000	PP = 50, HDPE = 60 and LDPE = 30	The recycled plastic wastes were pre-soaked in the asphalt for 60 min at 160 °C before mixing to ease the blending process.	[22]
N/A	Recycled polyethylene called PE1 and PE2	PE1 = 132.3 PE2 = 129.1	N/A	N/A	Trademark bitumen BNK 40/180	PE1= 5.4 PE2 = 3.9	180	420	180	When recycled polyethylene was introduced into the asphalt binder, the plasticity interval and viscosity of the asphalt binder increased significantly. The compatibility of asphalt binder and recycled polyethylene depends on the bulk properties of the polymer used. The Two mechanisms of the modifying action of recycled polyethylene were revealed: 1. Polyethylene with a higher melting temperature and narrow crystalline melting range does not interact with the dispersion medium of asphalt binder and serves as an inert filler, increasing the amount of disperse phase. 2. Polyethylene with a lower melting temperature and wide crystalline melting range combines with asphalt binder better.	[30]
Waste PET-based drinking bottles	Waste Polyethylene Terephthalate (PET)	PET = N/A	PET = 254 PET–TETA = <122, and PET–EA = 235	PET = Shredded/≤ 10, and CR = Powder/< 0.0232	60/70	CR = 18, PET–TETA = 2, and PET–EA = 2	180	3500	CR = 60, and 18CRMA2PET–TETA = 30 and 18CRMA2PET–EA = 30	The overall performance of rubberized bitumen improved when it was modified with treated waste PET. However, the incorporation of PET–TETA to modify the rubberized bitumen showed a significant increase in fatigue resistance. Whereas incorporation of PET–EA exhibited better resistance to permanent deformation.	[14]

^1^ Melting temperature, ^2^ Mixing temperature, ^3^ Reference.

## 2. Materials and Methods

### 2.1. Materials

#### 2.1.1. Asphalt Cement Properties

In this study, unmodified (virgin) asphalt cement (AC) PG 58–28 was used. Similar AC was used in previous studies at the University of Waterloo. The fundamental binder properties are presented in Table 2 [31]. The unmodified PG 58–28 was blended with MPP additives at concentrations of 2, 4, and 8 percent (by binder weight) to produce a total of four different binders. Each binder was tested at the appropriate test temperatures as designed by AASHTO standards. Prior to modification, binders were required to meet the AASHTO M320 standards. A series of DSR tests were carried out, following AASHTO T315, on the asphalt binders to characterize and determine the effect of MPP on the modulus at the high and intermediate performance grade temperatures.

#### 2.1.2. Multi-Layer Plastic Packaging

The MPP used in this project contained Polyethylene (PE), Polyester (PET), Nylon (NY), and Metallized Polyester (METPET) with different structures (PE-PETMET-PET, PE-PET, PE-NY-PET). The MPP was provided by Peel Plastic Products Ltd. In addition to the MPP material, a recycled Low-density polyethylene (LDPE) was also investigated in this project as the second alternative for recycled plastic material. Table 3 illustrates the typical physical properties of the film materials used. After computing the total volume and mass for each bag, total bag structure percentages were calculated, and thus the total percentage composition of each polymer type was obtained, as summarized in Table 4.

#### 2.1.3. The MPP Additives Preparation

**Figure 1:**
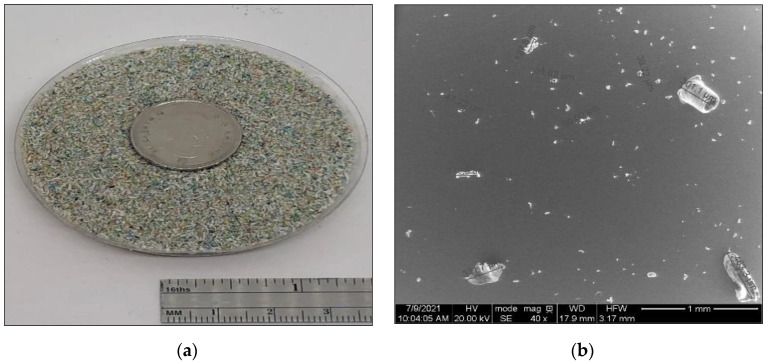

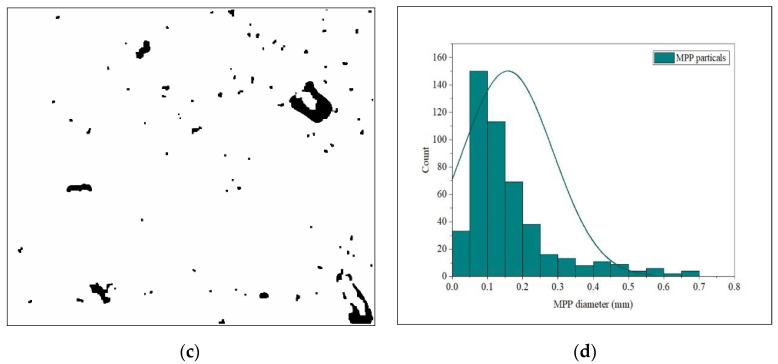
(**a**) MPP powdered form, (**b**) ESEM image of MPP at 40× magnification (1 mm), (**c**) MPP size analysis using ImageJ, and (**d**) MPP particle distribution (mm).

**Figure 2:**
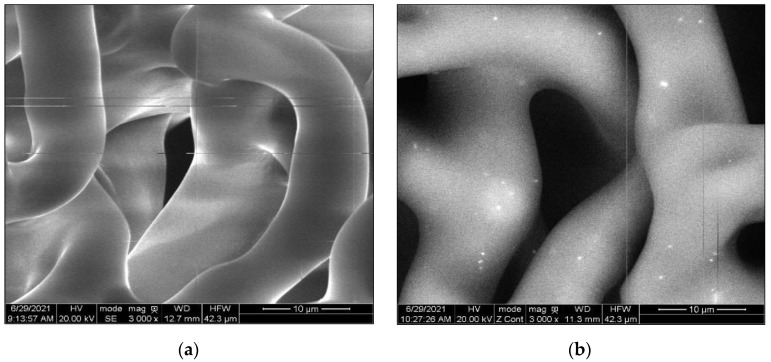
ESEM images of (**a**) unmodified PG 58–28 binder and (**b**) Blend−8 at 3000× magnification (10 µm).

To prepare MPP powder additives, a Micro-Cut electric shredder was used to shred the MPP into small particles (2–6 mm). The shredded material was ground to powder using an Electric Grain Mills Grinder (Ultra Grinder Machine) and then sieved to determine the grain size distribution of the MPP powder. The particle size distribution of the MPP powder to be added to the asphalt binder was between 0.075 to 0.595 mm, see Figure 1. ESEM was utilized to ensure the MPP powder size meets the AASHTO and ASTM particle size recommendation below 250µm in the modified binder before the DSR test. The test results showed that the MPP additive powder size was reduced after binder modification. Most of these particles melted and integrated into the asphalt binder. Particles with higher melting points did not melt, but their size was reduced to a size below the recommendation of AASHTO T 315 and ASTM D7175 standards (less than 250 µm), as shown in Figure 2 below.

#### 2.1.4. The MPP-Modified Asphalt Preparation

The virgin binder used in this experiment was PG 58–28. All MPP and LDPE modifications were performed at 2%, 4%, and 8% by the total weight of asphalt cement. Initially, the selected concentrations were determined based on the literature. Based on Table 4, the relative proportion of PET in each plastic product is different between PE-METPET-PET and PE-NY-PET. The smallest addition (e.g., 2% of PE-NY-PET) reflects the relatively higher proportion of ‘pure’ PET in that product, where it represents 6% compared to 5% in the PE-METPET-PET; thus, the 2 and 4% additions of PE-NY-PET and PE-METPET-PET were selected to produce blends with similar overall PET content. The 8% addition for both plastics was selected as a “ceiling” based on previous research that tended not to exceed 10% by weight of the binder. Table 5 shows the identified blends’ ID and their selected percentage additives for each blend used in this study. The mixing procedure was performed in two steps:

- Step 1: the hot asphalt binder was mixed with the additives using a stirring bar until the additives and asphalt binder produced a homogenous blend.
- Step 2: a high shear mixer was used to enhance the homogeneity of the blend at a temperature of 175 °C (±5 °C) at a rotational speed of 3500 rpm for one hour.

The mixing temperature was selected with respect to the thermal properties obtained from the DSC and TGA tests. The blend is a representative mix by mixing all the MPP types, as shown in Table 4. This blend will be further analyzed in the result and discussion section.

### 2.2. Experimental Methods

This section briefly describes the various laboratory experiments performed in this research investigation. A summary of the experiment’s objectives and methods is stated herein. Detailed steps of these experiments could be obtained from the AASHTO or ASTM standard testing methods. The laboratory experiments were classified into experiments to perform thermal analysis, which is associated with a material-dependent response when heat is supplied to asphalt samples, and others for rheological analysis, which is associated with the flow and deformation characteristics of asphalt binder.

#### 2.2.1. Thermal Analysis

##### Differential Scanning Calorimetry

The Differential Scanning Calorimetry (DSC) Q2000 is utilized to measure the thermal properties of MPP additives. The DSC laboratory testing was performed on the MPP and LDPE samples following ASTM D3418 to determine the melting, crystallization, and glass transition temperature information. Samples (weight range 10 to 20 mg) were subject to two cycles of cooling and heating. Each cycle started by maintaining the sample temperature at −90 °C for 2 min, followed by increasing the temperature to 260 °C and maintaining the temperature for 2 min. The heating and cooling rates were conducted at a rate of 10 °C per minute.

##### Thermogravimetric Analysis

Thermogravimetric Analyzer (TGA) is utilized to measure the amount and rate of change in weight of MPP and MPP-modified asphalt samples. TGA measures the weight change of materials from ambient temperatures up to 1000 °C and detects phase changes resulting from decomposition, oxidation, or dehydration. Results of TGA are correlated to the thermal stability of both the MPP additives and asphalt cement samples used in this study. The heating rate used in the TGA was 10 °C/min.

#### 2.2.2. Morphology Observation

##### Environmental Scanning Electron Microscopy

Environmental Scanning Electron Microscopy (ESEM) is carried out to determine the MPP size before and after the blending process at a microstructural scale. In addition, ESEM is utilized to ensure random and homogenous distribution of the MPP particles in the asphalt cement prior to measuring the storage stability. The ESEM is used to assess the presence of segregation between the asphalt cement and MPP. ESEM setting was performed according to Mikhailenko et al.’s [32] recommendations.

#### 2.2.3. Physical Properties

##### Rotational Viscometer

The viscosity of unaged asphalt samples is typically measured using the Rotational Viscometer (RV). The Strategic Highway Research Program (SHRP) stated a maximum viscosity of 3 Pa.s at 135 °C for workability purposes [33]. The binder’s viscosity at mixing temperature is specified at 0.17 Pa.s ± 0.02. The binder’s viscosity at compaction temperature is specified at 0.28 ± 0.03 Pa.s [34]. The ratio between the applied shear stress and the shear rate is referred to as viscosity. Viscosity measures the resistance of liquid material to flow (measured in Pascal-second, Pa.s). The test protocol used, according to AASHTO T 316 [34], specifies using an SC4-21 spindle at test temperatures of 135 °C, 150 °C, and 165 °C, and with speeds of 20, 50, and 100 rpm, respectively.

#### 2.2.4. Rheological Performance

##### Dynamic Shear Rheometer

Dynamic Shear Rheometer (DSR) is performed to measure the binder’s complex shear modulus (G*) and phase angle (δ°). G* is the parameter indicating the binder’s total resistance to deformation when frequently shared, and the δ° measures the delay between the applied shear stress and the resulting shear strain. The phase angle shows the range of the behavior of binder cement material from 0° (perfectly elastic material) to 90° (perfectly viscous material). Temperature and loading frequency has a direct effect on the G* and δ° values. Virgin and MPP-modified asphalt binders were tested following the AASHTO 315 to determine the G* and δ° [35].

##### Multiple Stress Creep Recovery

Multiple Stress Creep Recovery test is performed to measure elastic recovery, non-recoverable compliance (J_nr_), and percentage recovery in virgin and MPP-modified asphalt samples at high temperatures. The test is performed based on (AASHTO MP19 and AASHTO M332) using the DSR equipment [36]. The testing mechanism includes the application of a specific load for one second followed by a 9-s rest period. The experiment starts with ten low-stress cycles (0.1 kPa) followed by ten high-stress cycles (3.2 kPa). The outcome of the test is used to estimate the permanent deformation of the asphalt cement by using the non-recoverable compliance (J_nr_) to determine the corresponding traffic range in Equivalent Single Axle Loads (ESALs), as illustrated in Table 6. High creep resistance is reflected by achieving the low value of (J_nr_), and high value of R. Based on recommendations stated in AASHTO MP19, the J_nr_ value corresponding to a 3.2 kPa load is used to determine the presence of a sufficient amount of elastomer in the modified asphalt. Finally, the standard curve can be developed to show the presence of a sufficient amount of elastomer in the modified asphalt. In this study, the MPP R-value did not exceed the standard curve due to its low elasticity.

The standard MSCR curve is the reference point to evaluate the elasticity of binders. If J_nr_ is higher than the MSCR curve, this indicates the binder has high elasticity, such as elastomeric polymers. If the J_nr_ results are below the reference MSCR curve, this indicates the binder has low elasticity, such as plastomeric polymers.

##### Linear Amplitude Sweep

The linear amplitude sweep test (LAS) is used to determine the fatigue damage resistance of the asphalt cement according to AASHTO TP101. The LAS test uses cyclic torsion to increase strain amplitude steadily. Cyclic torsion results in the failure of the asphalt cement sample. The LAS test contains two tests, a frequency sweep and an amplitude sweep test. The frequency sweep test is performed by applying a constant strain of 0.1% and frequencies ranging from 0.2 to 30 Hz. The frequency sweep data is used to calculate the damage analysis “alpha” parameter. The linear amplitude sweep is performed by applying a linearly increasing strain within the range of 0–30% strain at a frequency of 10 Hz. The Viscoelastic Continuum Damage (VECD) model was used to determine the fatigue life of the asphalt binder as a function of strain, according to Hintz et al. [37]. Equation (1) was used to calculate the fatigue life of the modified binders. The LAS test was performed on the RTFO-aged samples at an intermediate temperature to evaluate fatigue damage resistance by conducting the number of cycles to failure (N_f_). The N_f_ at 50% reduction from the initial modulus was selected to determine the peak stress failure as recommended by AASHTO TP101 [38].

(1) $$N_{f}=A\;{\left(\gamma_{max}\right)}^{B}$$

where *Nf* is the number of cycles to failure, $\gamma_{max}$ is the maximum applied shear strain, and the parameters *A* and *B* are constants determined through the material characteristics.

##### Complex Shear Modulus under Frequency Sweep

The frequency sweep test applies a 0.5% strain rate at four low test temperatures of 5 °C, 15 °C, 25 °C, and 35 °C and uses an 8 mm parallel plate with a 2 mm gap. The high testing temperatures are 46 °C, 58 °C, 64 °C, 76 °C, and 82 °C and the experiment is performed using 25 mm parallel plate with a 1 mm gap and loading frequency sweep at 5% strain. The complex shear modulus (G*) and the phase angle (δ°) values were obtained for each MPP-modified asphalt sample. The G* and δ° were used to develop the master curve. A 2S2P1D model parameters were used to model the viscoelastic response of the MPP samples based on the recommendations of Nur et al. [39]. The data from the DSR test were modified by time-temperature superposition shift factors using the William–Landel–Ferry (WLF) Equation (2) to develop the master curve for the MPP-modified asphalt samples.

(2) $$loga_{t}=\frac{-C_{1}\left(T-T_{ref}\right)}{-C_{2}+\left(T-T_{ref}\right)}\;$$

where $T_{ref}$ is the reference temperature and $C_{1}$ and $C_{2}$ are referred to as material constants.

The black space diagrams present complex modulus vs. phase angle. Black space diagrams were also developed using the frequency sweep test data.

#### 2.2.5. Storage Stability

Storage stability in modified binders could be analyzed using several techniques. In this study, the separation ratio was used to evaluate storage stability. The storage stability test, commonly known as the cigar tube test (CTB), was used to determine the separation tendency of MPP-modified asphalt following the ASTM D 7173–11 [40]. The CTB test uses an aluminum tube (25 mm diameter and 140 mm height) to hold the material during storage. The storage stability test was performed by filling tubes with 50 ± 0.5 g of MPP- modified asphalt binders. The tubes were sealed and stored vertically in an oven at 163 ± 5 °C for 48 h. The tubes were transferred to a freezer at −10 ± 1 °C for a minimum of 4 h until the material was completely solidified. Individual tubes with the solidified binder were split into three equal specimens, and the center specimen was discarded. The top and bottom specimens were used to determine the rheological properties of the binder, including G* complex modulus and phase angle δ°, and to test the morphology of the binder. Low compatibility between asphalt binders and polymers leads to a reduction in the storage stability of the modified binder. Low compatibility could result from differences in molecular structure, density, molecular weight, and viscosity of the polymer and asphalt components [41].

##### The Separation Ratio

In this study, the separation ratio was used to evaluate storage stability by applying Equations (3) and (4). The control, all MPP and LDPE modified binders were examined using DSR tests to obtain the rheological properties parameters ($G^{*}\;and\;\delta {}^{\circ})$ at 58 °C. Separation ratios $R_{s}\left(G^{*}\right)and\;R_{s}\left(\delta {}^{\circ}\right)$ were calculated using equations (3) and (4). (NCHRP) 9–10 report recommended the values of the separation ratio within 0.8 to 1.2 to avoid binder separation [42].

(3) $$R_{s}\;\left(G^{*}\right)=G^{*}\;separation\;ratio=\frac{{\left[G^{*}\right]}_{top}}{{\left[G^{*}\right]}_{bottom}}$$

(4) $$R_{s}\left(\;\delta {}^{\circ}\right)=\delta \;separation\;ratio=\frac{{\left[\delta {}^{\circ}\right]}_{top}}{{\left[\delta {}^{\circ}\right]}_{bottom}}$$

## 3. Results and Discussion

### 3.1. Differential Scanning Calorimetry

Table 7 summarizes the maximum endothermic peak integration, which refers to the melting points of all MPP and LDPE additives. The mixing temperature for all MPP and LDPE materials in this study was selected at 175 °C ± 5 °C to ensure the POLY and LDPE material enter the melting phase and blend with the liquid asphalt cement. The remaining components of the MPP materials used in this project (PET, NY, and METPET) represent less than 15% of the total MPP weight. These materials were treated as fillers due to their high melting points.

### 3.2. Thermogravimetric Analysis

**Figure 3:**
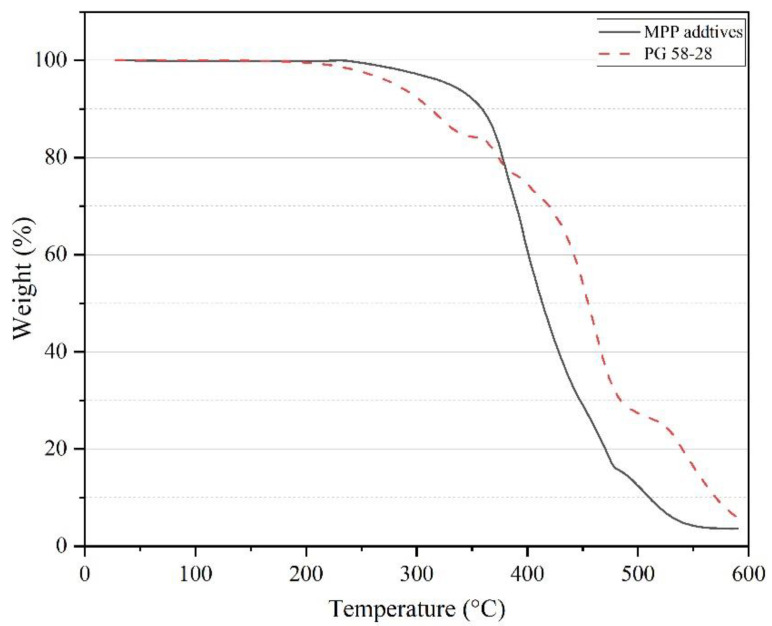
TGA curves of MPP additives and PG 58–28.

The magnitude and rate of change of MPP weight and asphalt cement samples were analyzed using TGA. Figure 3 demonstrates the thermogravimetric curve of the virgin PG 58–28 binder and MPP additives from 25 °C to 600 °C. Both MPP additives and PG 58–28 showed high thermal degradation temperatures. The degradation was limited to 5% for MPP additive up to 320 °C and almost 10% for PG58–28. The significant degradation occurred after 400 °C for the MPP additives, while the PG58–28 started rapid degradation around 320 °C. The selected mixing temperature of 175 ± 5 °C prevents the occurrence of significant thermal degradation to the mix.

### 3.3. Rotational Viscometer

**Figure 4:**
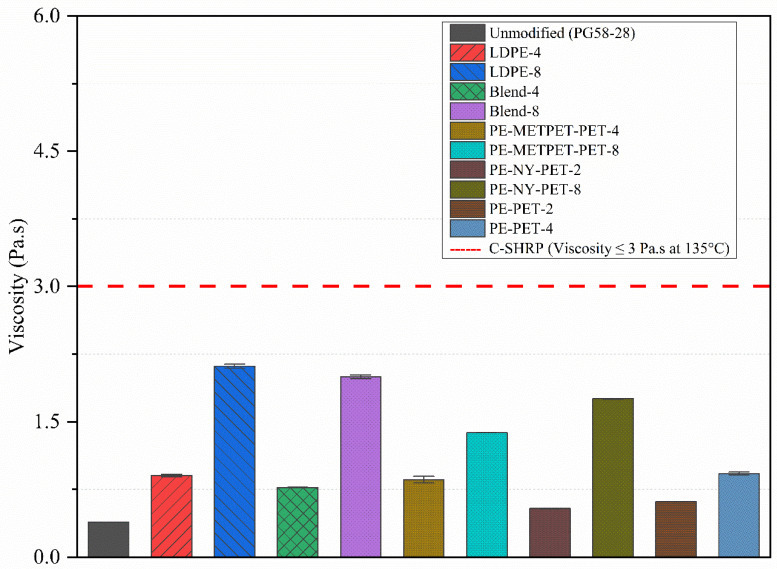
SHRP requirement 3.0 Pa.s at 135.

The viscosity of modified asphalt binders using MPP and LDPE was measured to ensure the modified binder met the Superpave binder specification. Recommendations of Superpave specification stated that binder viscosity should not exceed 3 Pa.s when measured at 135 °C. Figure 4 presents the viscosity results measured at 135 °C. All the dosages 2%, 4%, and 8% showed a noticeable increase in the viscosity of all MPP-modified asphalt binders and LDPE-modified binders. However, all MPP-modified binders did not exceed the Superpave requirement. The viscosity of 2% MPP-modified binders was 50% higher than that of the control sample. The increase in viscosity was 100% higher in the 4% MPP-modified binder and 400% higher in the 8% MPP-modified binder.

**Figure 5:**
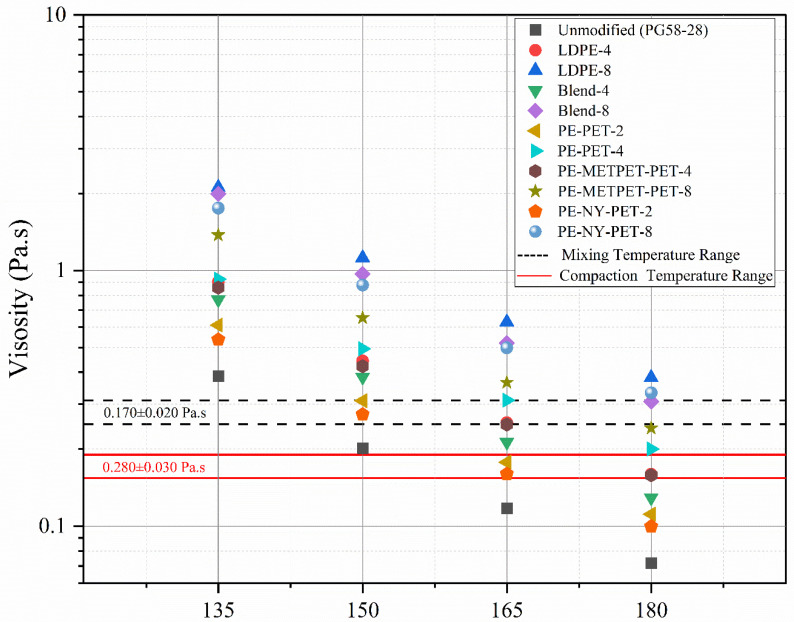
Temperature-viscosity curves of MPP and LDPE asphalt binders.

Multiphase systems that occur when asphalt blends with polymers can lead to complex inter-molecular friction [43]. This complexity can lead to an increase in the applied shear stress, consequently increasing viscosity. In this study, the increase in viscosity values could be attributed to the effect of the non-dissolved particles and the difference in molecular structure, weight, and density of MPP additives [22]. To ensure the increment dosage showed the same trend even at different temperatures, changes were measured at 150, 165, and 180 °C, as presented in Figure 5. The increase in the dosage subsequently increased the calculated mixing and compaction temperature range of all the modified binders, as presented in Figure 5. The increase in viscosity of modified binders resulted in an increase in mixing and compaction temperature ranges starting from 5 to 30 °C. This increase in viscosity could increase the resistance to high-temperature deformation. However, it has a high cost due to increased energy consumption and the production of additional greenhouse gases.

### 3.4. Rheological Characterization

#### 3.4.1. Dynamic Shear Rheometer

##### Rutting and Fatigue Parameters

**Figure 6:**
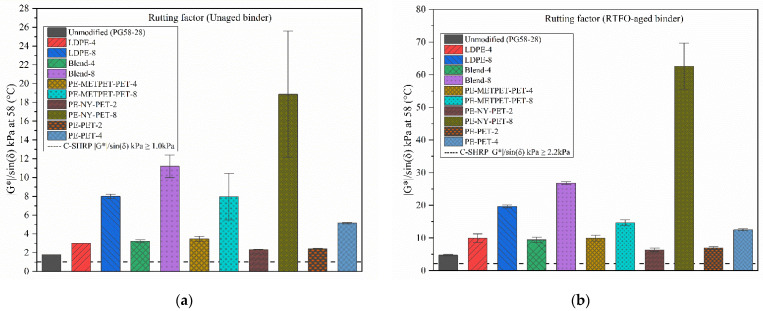
Rutting G*/sin(δ°) parameter for (**a**) unaged and (**b**) RTFO-aged binders.

The rheological properties of asphalt binders were characterized using the DSR test according to AASHTO M320 and M332. Superpave reported that G*/sin(δ°) factor for unaged and RTFO-aged asphalt binder samples indicates the potential resistance to permanent deformation at high temperatures. In this study, the G*/sin(δ°) increased by adding MPP and LDPE additives to the virgin asphalt, as shown in Figure 6. The addition of MPP or LDPE in any amount showed an increase of the G*/sin(δ°) for both unaged and RTFO-aged, modified binders when compared to the unmodified sample. The addition of 4% and 8% LDPE increased the G*/sin(δ°) from 1.8 to 3.0 KPa and 8.0 KPa, respectively. The addition of 4% and 8% MPP resulted in a higher increase of G*/sin(δ°) compared to the LDPE. The 8% MPP binder exhibited G*/sin(δ°) increase for unaged and RTFO-aged samples by more than 400% compared to the control binder. Binder modification using 8% PE-NY-PET resulted in an increase of G*/sin(δ°) from 1.8 KPa to 18.9 KPa and 62.6 KPa in unaged and aged binder samples, respectively. This noticeable change occurred due to the high elasticity and tensile strength of the NYLON.

The noticeable increase in G*/sin(δ°) through LDPE and MPP binder modification indicates a change in the high-temperature grade of unaged and RTFO-aged samples. Therefore, LDPE- and MPP-modified binders would have high resistance to rutting at temperatures exceeding the virgin binder’s PG high temperature. For example, the 8% MPP modified binders exhibited a shift in the high-temperature grade up to 100 °C while the virgin binder maximum high Superpave temperature for the virgin binder was 82° C according to Table 8.

**Figure 7:**
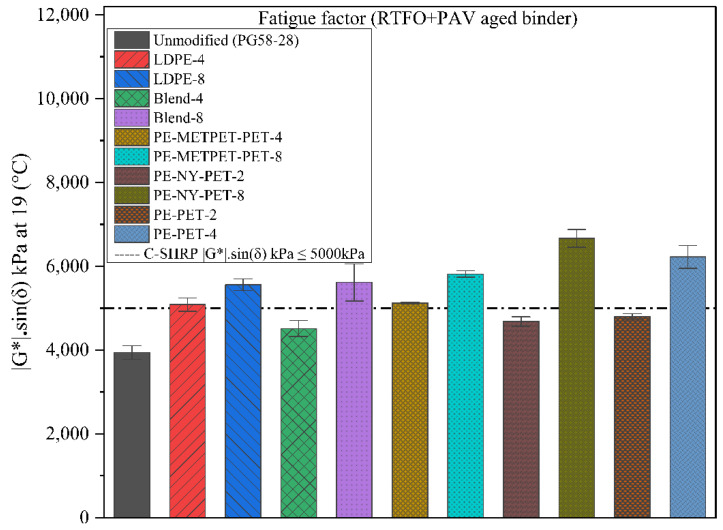
Fatigue parameter G*sin(δ°) for RTFO + PAV aged binders.

Figure 7 illustrates the difference between the fatigue parameter G*sin(δ°) for various virgin and modified binders. Generally, G*sin(δ°) reflects the linear viscoelastic properties of the MPP-and LDPE-modified binders. However, several studies concluded that the G*sin(δ°) fatigue parameter does not accurately measure the correlation between the asphalt binder fatigue resistance and pavement resistance to fatigue cracking as several parameters contribute to the mixtures fatigue resistance in addition to the binder’s G*sin(δ°) [44,45,46,47,48]. In addition, the weak correlation between the binder’s G*sin(δ°) and the mixture’s fatigue cracking resistance is that G* and δ° are measured within the linear viscoelastic region. This region does not represent the actual variety of strains or stresses that occur in the nonlinear viscoelastic region of pavement loading. In other words, the pavement response in the higher stresses and strains exceeds the linearity of the viscoelastic region.

Superpave specification (AASHTO M 320) concluded that higher fatigue resistance of asphalt binder could be obtained using a lower G*Sin δ° parameter. Figure 7 presents the G*sin(δ°) of all virgin and modified binders. All MPP and LDPE-modified binders exhibited an increase in G*sin(δ°) values compared to the virgin binder. This concludes that MPP and LDPE binder modification would potentially result in a reduction in the binder’s resistance to fatigue cracking within the linear viscoelastic material behavior. MPP- and LDPE-modified binders with a high percentage (exceeding 4% modification) exhibited G*sin(δ°) results that exceeded the Superpave maximum threshold of 5000 kPa at 19 °C. Since the G*sin(δ°) fatigue parameters cannot be used as an accurate performance measure to evaluate binder resistance to fatigue cracking and HMA resistance to fatigue cracking [42,43,44,45], these results are considered an indicator of the fatigue performance, but further testing on the HMA mixtures will be performed to develop a robust and comprehensive conclusion about the impact of MPP and LDPE binder modification on the fatigue resistance.

The intermediate testing temperature of the virgin binder was 19 °C. The DSR results indicated that the intermediate temperature of MPP- and LDPE-modified binder exceeded 22 °C. This increase in the intermediate temperature is a direct result of the MPP- and LDPE-binder modification. Therefore, the MPP- and LDPE-modified binders should be tested at the intermediate temperature determined by the DSR rather than the recommended temperature based on the grade of the virgin binder. To set a consistent testing reference temperature in this project, all binder samples (virgin and modified) were tested at 19 °C as the intermediate testing temperature.

#### 3.4.2. Multiple Stress Creep Recovery

**Figure 8:**
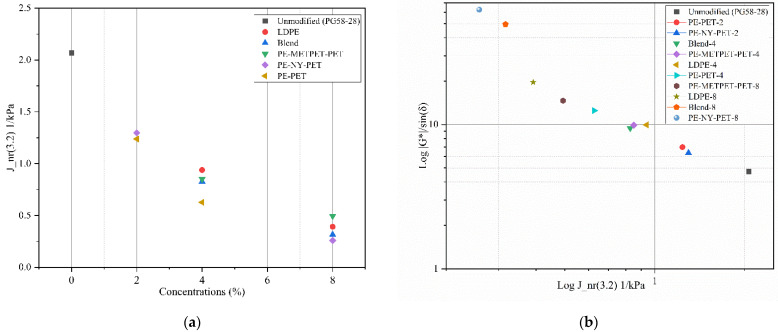
(**a**) J_nr_ (3.2 kPa) vs. additive concentration (%); (**b**) Log J_nr_ 3.2 vs. Log G*/sin(δ°).

Multiple Stress Creep Recovery (MSCR) test was performed according to AASHTO T350 to determine the following parameters J_nr_ 3.2, percent recovery, and J_nr_ difference. The test was performed at 58 °C based on Ontario’s climatic conditions [49]. Figure 8a presents the noticeable changes in J_nr_ 3.2 with the increase of all MPP and LDPE additives. The rate of change of J_nr_ 3.2 increased consistently by adding the binder modifiers leading to an increase in the binder modulus. An increase in binder modulus could be interpreted by the reduction of binder strain under traffic loads within the linear elastic range of the material properties when preventing permanent deformations. Figure 8b presents the result of J_nr_ 3.2 versus the G*/sin (δ°). The figure concludes an inverse correlation that binder resistance to permanent deformations increases (i.e., increase in G*/sin (δ°)) by decreasing J_nr_ 3.2 (i.e., higher MPP and LDPE concentrations).

**Figure 9:**
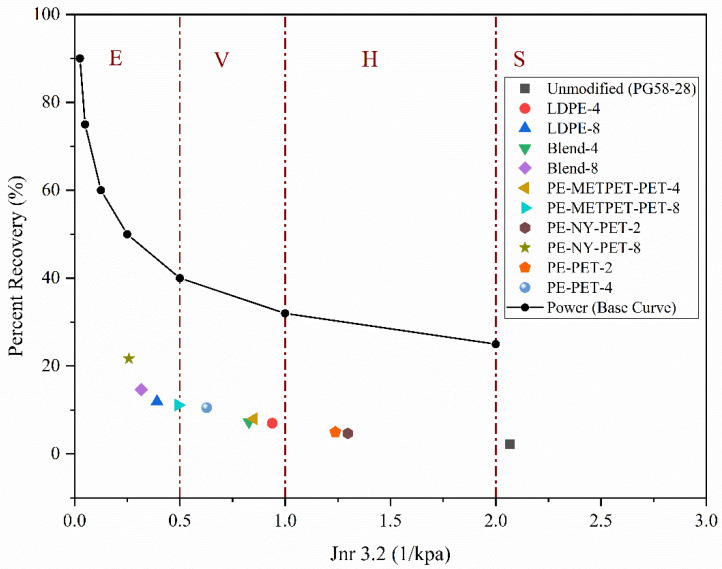
Relationship between R and J_nr_ for MPP- and LDPE-modified binders.

Figure 9 demonstrates the relationship between the percentage recovery and non-recoverable compliance for virgin, MPP-modified, and LDPE-modified binders. Modified binders exhibited an improvement in traffic grades compared to virgin binders. The increase of MPP and LDPE modifiers from 2% to 8% led to a traffic upgrade in MSCR traffic grades from the standard traffic “S” to Extremely Heavy traffic “E”. The percent recovery results of the blends that contain NYLON (PE-NY-PET and Blend-8) had higher percentage recovery (i.e., reflecting more elastic behavior). The percentage recovery increased from 2.22% for the virgin binder to 14.68% and 21.68% for Blend-8 PE-NY-PET-8, respectively. This indicates higher resistance to permanent deformation of the modified binders.

#### 3.4.3. Linear Amplitude Sweep

**Figure 10:**
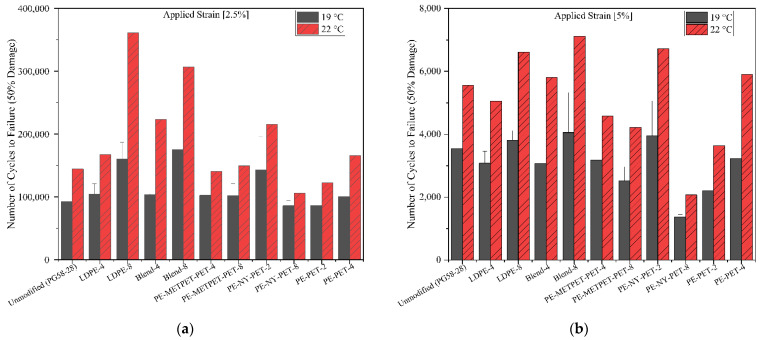
LAS results of MPP-LDPE-modified binders at 19 °C and 22 °C (**a**) 2.5% strain; (**b**) 5% strain.

The LAS test is used to evaluate the fatigue damage resistance of the asphalt binder under standard AASHTO TP101. LAS is performed to determine the number of cycles to failure under fatigue cycles (N_f_). The LAS test was performed at 19 °C and 22 °C and two strain levels (2.5% and 5%) representing the thick and thin pavements, respectively. Figure 10 presents the results obtained using a viscoelastic continuum damage (VECD) model at 50% damage levels. The results present the difference in LAS for all MPP- and LDPE-modified binders versus the virgin PG 58–28 binder. LAS performed at 19 °C, and 2.5% strain showed a slight increase of 7% in N_f_ for PE-NY-PET-8 and PE-PET-4 modified binders compared to the virgin binder. The LDPE-4, Blend-4, PE-METPET-PET-4, and Blend-8 modified binders exhibited a 10% increase in N_f_ compared to the virgin binder. PE-PET-2 was the only modified binder that exhibited lower N_f_ compared to the virgin binder. Other modified binders exhibited more significant increases in N_f,_ like PE-NY-PET-2, LDPE-8, and Blend-8, which increased by 50%, 70%, and 230%, respectively, compared to the virgin binder. LAS results at 22 °C generated similar trends as testing at 19 °C except that PE-METPET-PET-4 and blend-8, PE-NY-PET-8, and PE-PET-2 modified binders showed a smaller percentage of increase in N_f_ compared to the percentage of increases obtained at 19 °C.

The mobility of the polymer chains during fatigue loading results in early-stage brittle failure [41]. This phenomenon is associated with high molecular weight (Mw) and densifier-packed molecules. In contrast, low Mw increases chain mobility, which leads to an increase in resistance to shear yielding and ductile failure. Low Mw binders are expected to exhibit more N_f_ compared to binders with high Mw. The results of N_f_ for 8% LDPE at 5% strain level show an increase of 7% and 19% at 19 °C and 22 °C, respectively, compared to the virgin binder. On the contrary, a reduction of N_f_ is noticed in PE-NY-PET-2–8 and PE-PET-2-4 by comparing the N_f_ of modified binders to the virgin binder. This is because ductile-brittle transition occurs at an early stage with mixes containing higher PE concentration. This is justified by the high Mw of the PE and densely packed molecules [41]. Finally, the LDPE-8 and Blend-8 characterized by low molecular weights exhibited an increase in the N_f_ compared to other mixes.

Results of the LAS test indicated that most of the modified blends exhibited an increase in N_f_ at 2.5% and 5% strain levels compared to the virgin binder. Since the LAS test considers the nonlinear viscoelastic behavior of asphalt binders, this experiment would offer a more accurate appreciation of the resistance to fatigue cracking compared to the fatigue factor G*sin(δ°) [50]. However, the discrepancy between the two tests confirmed the absolute need to use the mixture performance tests as a reliable evaluation when it comes to evaluating fatigue performance. Moreover, it confirmed the conclusions stated by Deacon et al. [44] that fatigue response in the pavement structure cannot be determined through testing asphalt binder only. Several factors significantly contribute to pavement fatigue performance, including mix characteristics, pavement structure, traffic, and environmental status [51].

#### 3.4.4. Complex Shear Modulus under Frequency Sweep

**Figure 11:**
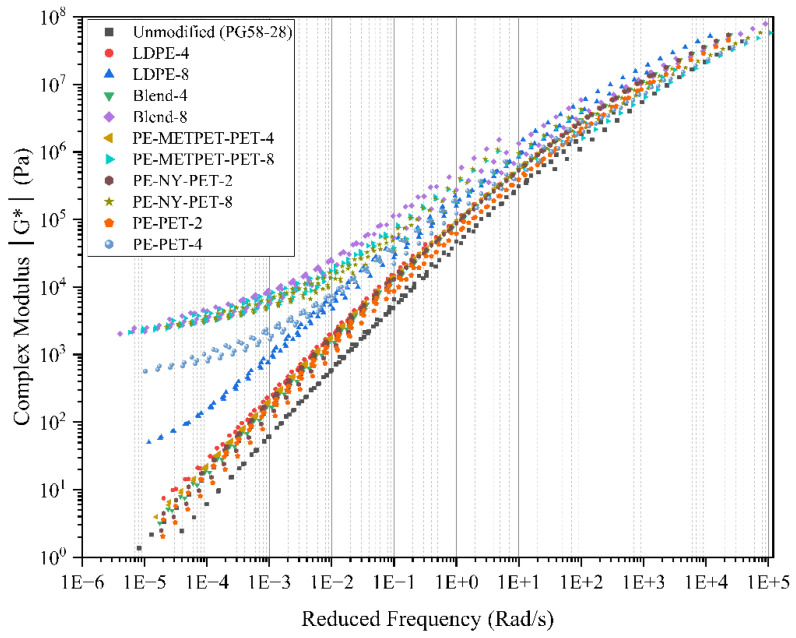
Modulus G* master curves for all MPP and LDPE modified asphalt original binders.

The experimental matrix in this project included measuring the complex shear modulus and the phase angle δ° (◦) at a standard range of temperatures and frequencies for control virgin binder, MPP-modified, and LDPE-modified binders. The complex shear modulus includes a relative component of the elastic and viscous response to loading. Master curves were developed for all MPP- and LDPE-modified binders unaged samples. The results indicate a gradual increase of complex modulus at the high modified frequencies (representing low temperatures) and a reduction in modulus at low modified frequencies (representing high temperatures). The complex modulus significantly increased at all modified binders compared to the virgin binders, as expected from the MSCR test results. For example, MPP-modified binders, with 8% MPP, have shown a significant increase in the complex modulus that exceeded that of the virgin binder up to 10 times (at certain frequencies and testing temperatures). The LDPE-modified binder at an 8% modification rate has shown a more modest increase of complex modulus up to 3.5 times at certain frequencies and testing temperatures compared to the virgin binder. The increase in the stiffness aligns with the findings of the MSCR test and confirms the strong potential of improving the permanent deformation resistance using MPP- and LDPE-modification. The temperature range between 15 °C and 35 °C showed a change by +50%, +70%, and 100% of complex modulus for 2, 4, and 8% MPP- and LDPE- modified binders, respectively. Finally, at a low testing temperature (5 °C), the change of the complex modulus was less than 30% for 8% binder modification and less than 10% for 2% binder modification compared to the virgin binder, as presented in Figure 11. Therefore, a low modification percentage of MPP additives could maintain the low-temperature performance with a mild impact on the high-temperature properties. High binder modification that exceeds 4% could be used to resist higher stresses and severe temperature conditions.

**Figure 12:**
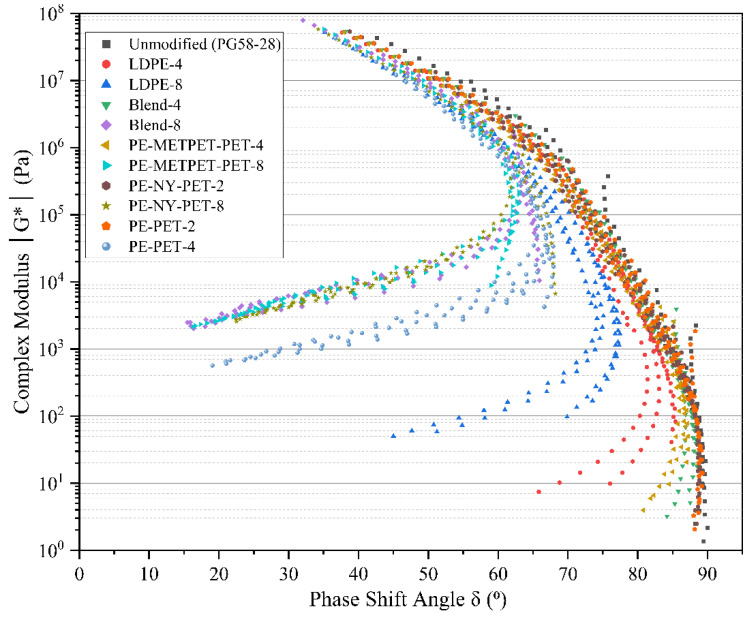
Black space diagrams for MPP- and LDPE-modified asphalt original binders.

According to Airey [52], black diagrams can provide a quick assessment of the simple thermo-rheological behavior of asphalt cement and any critical changes in rheological outcome. Therefore, the black space diagrams were developed to study the thermo-rheological behavior of all MPP- and LDPE-modified binders based on the shear complex modulus versus the phase angle δ°, as displayed in Figure 12. The black space diagram results showed that there is an obvious shift in the phase angle values of all MPP- and LDPE-modified binders at high temperatures for binder modifications exceeding 4% compared to the virgin binder. The effect of adding any type of MPP or LDPE modification at or above 4% concentration substantially increases the elasticity of the binder, particularly at higher temperatures, and leads to a reduction of the phase angle by more than 50%. The decrease in the phase angle is due to the elastomeric nature of the plastic. The presence of plastic with a high percentage changes the modified binder toward elastic behavior, confirming the positive impact at the high-temperature permanent deformation resistance. This phenomenon has a direct link to the ability of the polymer modifier to form a continuous elastic network between asphalt cement and the modifier when dissolved in the asphalt blend [53]. The remaining MPP particles characterized by higher melting points can dominate the rheological behavior when the high binder modification percentage of the MPP additive is used. Similar behavior was found with modified asphalt using crumb rubber and polyethylene (PE) [54,55].

The Black space diagram results at the low temperatures show no obvious difference among all the modified binder blends with 4% or less of MPP and LDPE additives. Therefore, it can conclude that the presence of MPP and LDPE additives with 4% or less does not have a significant rheological impact at low temperatures. It would be beneficial to use a low percentage of MPP additives to maintain low-temperature performance.

### 3.5. Storage Stability

#### 3.5.1. The Separation Ratio

**Figure 13:**
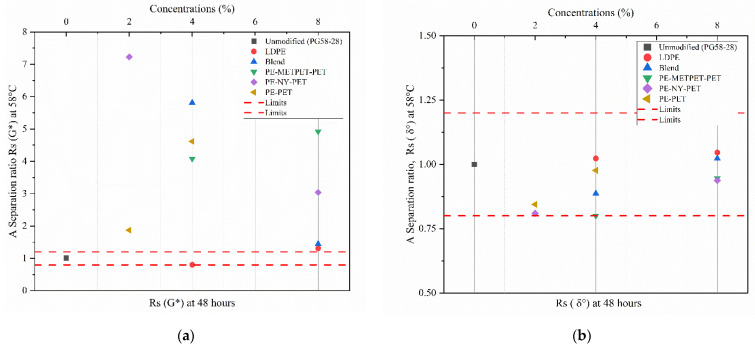
Separation ratio (Rs) (**a**) Rs based on (G*); (**b**) Rs based on δ°.

A common drawback of using polymers in asphalt modification is phase separation [16,56]. Several approaches have been used to assess phase separation. The tube test, which has a better simulation of the storage conditions of the asphalt binder, is one of these tests. According to (NCHRP) 9–10 report, the separation ratio must be within the recommended values of 0.8 to 1.2 to avoid binder separation [42]. The results of the separation ratio Rs based on G* are shown in Figure 13a. The presented results confirmed that storage separation would be a concern with all MPP and LDPE additives and at all concentrations.

However, the separation ratio Rs based on δ° (presented in Figure 13b) did not confirm the separation. Storage separation problems could not be identified through the determination of Rs based on δ° due to the DSR test limitations testing the modified binders. It was noticed that PE-NY-PET-8 and Blend-8 containing NY exhibited low separation compared to lower-concentration blends. LDPE-8 exhibited a high separation compared to LDPE-4. Roja et al. reported that the addition of polyethylene to asphalt binders tends to separate at high temperatures due to its non-polarity and non-aromaticity [42]. In this study, the presence of NY, METPET, and PET at high concentrations in MPP-modified blends resulted in a reduction in separation. This phenomenon is explained by the high polarity and aromaticity of NY, METPET, and PET materials [57]. This finding suggests that the results based on the G* and δ° are not the most accurate indicator to evaluate the storage stability of the modified binders. Therefore, examining the presence of the additive’s particles at the microstructure level of the storge samples would be recommended.

#### 3.5.2. Storage Stability Using ESEM

**Figure 14:**
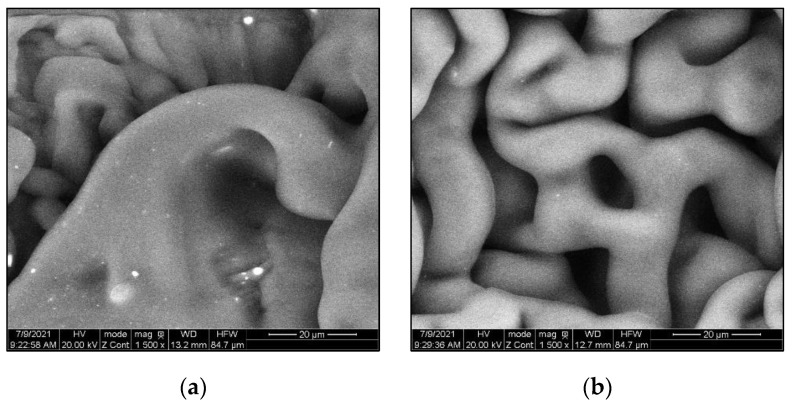

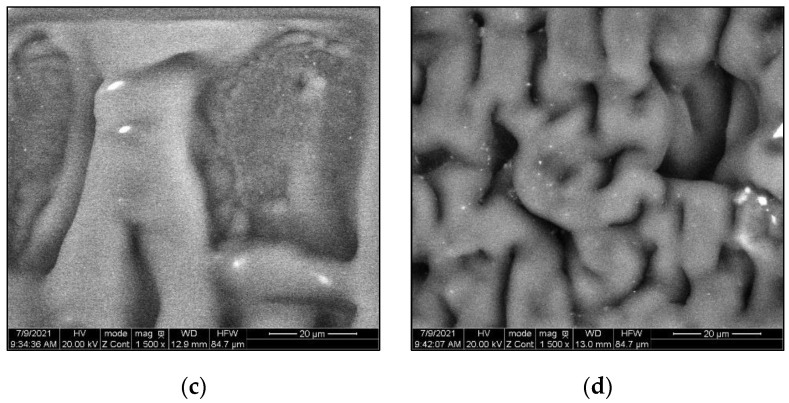
ESEM images of storage stability samples (Top and Bottom) at 1500× magnification (20 µm) (**a**) Blend-4 Top, (**b**) Blend-4 Bottom, (**c**) Blend-8 Top; (**d**) Blend-8 Bottom.

ESEM test was performed on the storage stability samples to observe how the particles at the top and bottom of the samples of the Blend-4 and -8 modified binders dispersed, as shown in Figure 14. The presence of the MPP particles at both top and bottom samples were notable, with a higher concentration of 8% in comparison to Blend-4. This finding aligns with the results of the separation ratio outcome, which confirms that MPP with different densities, polarity, and molecular weight can reduce the mobility of the polymer to migrate from the bottom to the top. This finding indicates that increasing the quantity of NY, PET, and METPET could reduce the phase separation and alleviate the storage stability concerns for asphalt with polymer modifiers. This finding demonstrates the need for a more in-depth investigation of the use of analytical techniques to evaluate the storage stability issue of MPP-modified binders.

## 4. Conclusions

The measurement of the virgin, MPP-, and LDPE- modified binders, including rheological, physical, morphological, thermal, and storage characteristics, were conducted in this study. The analysis assessed the impact of adding 2%, 4%, and 8% of MPP material and 4% and 8% of LDPE by the total weight of the asphalt cement. Based on the findings of laboratory investigation presented in this paper, the following conclusions are proposed:

- TGA results revealed multiple melting points ranging from 110 °C to 254 °C for all MPPs tested. Similarly, mass losses for asphalt samples and MPP additives, up to 320 °C, were negligible. These results, along with DSC, were used as criteria to determine the blending temperature.
- ESEM images showed that the MPP particle became significantly smaller after blending with the virgin asphalt and that most of the MPP additives were well integrated into the asphalt blend.
- The Brookfield viscosity test results confirmed that all MPP and LDPE additives would increase the viscosity and reduce the flow without exceeding the SHRP allowable limit (i.e., 3 Pa.s at 135 °C), resulting in acceptable workability performance.
- The rutting factor (G*/Sin δ°) exhibited an increase by adding the MPP and LDPE additives, which indicates the ability of asphalt binders to resist permanent deformation. Similarly, MSCR test results showed a noticeable reduction of J_nr-3.2_ with the increase of all MPP and LDPE additives, which is also an indicator of higher resistance to permanent deformation. Blends that contain NYLON (PE-NY-PET and Blend-8) had a higher percentage recovery, reflecting more elasticity compared to other mixes.
- The temperature-sweep test showed that all MPP and LDPE-modified binders exhibited a shift from predominantly viscous to elastic behavior when the testing temperature increased from 46 °C to 82 °C, at a 4% modification rate and higher, which is a strong indication of an improved rutting resistance.
- The results of the Linear Amplitude Sweep (LAS) test exhibited an increase in the number of cycles to failure under fatigue cycles (N_f_) in MPP- and LDPE-modified binders compared to the virgin binder. This indicates a potential improvement of fatigue cracking resistance in MPP-and LDPE-modified binders.
- MPP- and LDPE-modified binders would face some issues with storage stability. Due to their higher polarity, aromaticity, and density compared to PE, the blends that included NY, METPET, and PET have shown better stability and potential to reduce separation at high concentrations.

This study confirmed the feasibility of using MPP- and LDPE-modified binders to improve the fatigue and rutting behavior of asphalt pavements. Future work should include mixture tests to have a better understanding of the effect of MPP modifiers on the workability, rutting, fatigue cracking, and low-temperature resistance of asphalt mixtures.

## Figures and Tables

**Table 2:** Properties of asphalt [31].

Property	Test Method	PG 58–28
**Original Material**		
Ash Content, %	ASTM D2939–09	0.03
Viscosity (Pa.s), At 135 °C	AASHTO T316	0.266
G*/sin(δ°), kPa	AASHTO T315	1.18
**RTFO Residue**	AASHTO T240	
Mass Loss (%)	AASTHO T240	0.37
G*/sin(δ°), kPa	AASHTO T315	3.05
**PAV Residue**		
G*sin(δ°), kPa	AASHTO T315	3550
m-Value at Pass Temperature	AASHTO T313	0.358
Stiffness, MPa at Pass Temperature	AASHTO T313	187
m-Value at Fail Temperature	AASHTO T313	0.294
Stiffness, MPa at Fail Temperature	AASHTO T313	385
**True Grade**	AASHTO M320	59.4–31.4

*Complex shear modulus (G*).

**Table 3:** The typical physical properties of MPP and LDPE additives.

Material	Melting Temperature (Tm °C) ASTM D7138-16	Transition Temperature (Tg °C) ASTM D7138-16	Density (g/cm^3^)
Polyethylene (PE)	110–140	−120	0.9–0.95
Nylon (NY)	252–265	50	1.1–1.2
Polyester (PET)	240–255	75	1.4
Metallized Polyester (METPET)	240–255	75	1.4
Low-density polyethylene (LDPE)	110–140	−120	0.9–0.95

**Table 4:** The MPP and LDPE are based on the approximate total mass percentages.

Bag Structure	% PE	% METPET	%NY	% PET	Total
PE-METPET-PET	87	8	---	5	100
PE-PET	94	---	---	6	100
PE-NY-PET	86	0	8	6	100
Blend *	89	3	3	6	100
LDPE	100	---	---	---	100

* Blend is a representative mix by mixing all MPP.

**Table 5:** List of modified asphalt binder blends.

Asphalt Cement	Modifier	Modifier Tested (%)	ID in the Graphs
PG 58–28	None	0	Unmodified (PG58–28)
	LDPE	4, 8	LDPE-4 and LDPE-8
	Blend	4, 8	Blend-4 and Blend-8
	PE-METPET-PET	4, 8	PE-METPET-PET-4 and PE-METPET-PET-8
	PE-NY-PET	2, 8	PE-NY-PET-2 and PE-NY-PET-8
	PE-PET	2, 4	PE-PET-2 and PE-PET-4

**Table 6:** MSCR grades depend on J_nr_ values (AASHTO M332).

Designation Traffic Level	J_nr_ Value at 3.2 kPa−1	ESALs Million and Load Rate
“E” refers to Extremely high traffic loading	0.0–0.5	≥30 and <20 km/h
“V” refers to Very high traffic loading	0.0–1.0	≤30 or <20 km/h
“H” refers to High traffic loading	1.0–2.0	10–30 or 20–70 km/h
“S” refers to Standard traffic loading	2.0–4.0	≤10 and >70 km/h

Note: ESAL = equivalent single-axle load.

**Table 7:** Peak Integration by DSC for all MPP and LDPE.

MPP	Start	Onset	Maximum	Stop	Area
	°C	°C	°C	°C	J/g
PE-PET	64.12	113.35	119.69	165.21	73.76
	233.27	237.91	249.08	267.97	4.435
PE-NY-PET	42.56	112.97	119.48	158.13	69.78
	185.08	237.32	251.14	269.65	25.24
PE-METPET-PET	62.1	111.11	120.5	151.39	55.04
	215.75	241.03	253.04	278.08	16.31
LDPE	46.6	101.74	109.42	141.96	103.7

**Table 8:** High-temperature continuous grade of MPP and LDPE modified binders for Unaged and RTFO aged binders.

Asphalt Binder ID	Unaged Grading (°C)	RTFO-Aged Grading (°C)
Unmodified (PG58–28)	63	64
LDPE-4	67	70
LDPE-8	76	76
Blend-4	68	70
Blend-8	85 *	102 *
PE-METPET-PET-4	69	70
PE-METPET-PET-8	91 *	74
PE-NY-PET-2	65	67
PE-NY-PET-8	104 *	109 *
PE-PET-2	65	67
PE-PET-4	73	72

* Exceeds the maximum Superpave high temperature of 82 °C.

## Data Availability

All data used during the study appear in the submitted article.

## References

[1] (2019). Economic Study of the Canadian Plastic Industry, Markets and Waste: Summary Report to Environment and Climate Change Canada.

[2] Lu X., Isacsson U. (2000). Modification of Road Bitumens with Thermoplastic Polymers. Polym. Test..

[3] González O., Peña J.J., Muñoz M.E., Santamaría A., Pérez-Lepe A., Martínez-Boza F., Gallegos C. (2002). Rheological Techniques as a Tool To Analyze Polymer− Bitumen Interactions: Bitumen Modified with Polyethylene Polyethylene-Based Blends. Energy Fuels.

[4] Yildirim Y. (2007). Polymer Modified Asphalt Binders. Constr. Build. Mater..

[5] Satapathy S., Nag A., Nando G.B. (2010). Thermoplastic Elastomers from Waste Polyethylene and Reclaim Rubber Blends and Their Composites with Fly Ash. Process Saf. Environ. Prot..

[6] Kalantar Z.N., Karim M.R., Mahrez A. (2012). A Review of Using Waste and Virgin Polymer in Pavement. Constr. Build. Mater..

[7] Zheng Y., Shen Z., Cai C., Ma S., Xing Y. (2009). The Reuse of Nonmetals Recycled from Waste Printed Circuit Boards as Reinforcing Fillers in the Polypropylene Composites. J. Hazard. Mater..

[8] Hınıslıoğlu S., Ağar E. (2004). Use of Waste High Density Polyethylene as Bitumen Modifier in Asphalt Concrete Mix. Mater. Lett..

[9] Garcia-Morales M., Partal P., Navarro F.J., Gallegos C. (2006). Effect of Waste Polymer Addition on the Rheology of Modified Bitumen. Fuel.

[10] Casey D., McNally C., Gibney A., Gilchrist M.D. (2008). Development of a Recycled Polymer Modified Binder for Use in Stone Mastic Asphalt. Resour. Conserv. Recycl..

[11] Al-Hadidy A.I., Yi-Qiu T. (2009). Mechanistic Approach for Polypropylene-Modified Flexible Pavements. Mater. Des..

[12] Fang C., Yu R., Zhang Y., Hu J., Zhang M., Mi X. (2012). Combined Modification of Asphalt with Polyethylene Packaging Waste and Organophilic Montmorillonite. Polym. Test..

[13] Maharaj C., Maharaj R., Maynard J. (2015). The Effect of Polyethylene Terephthalate Particle Size and Concentration on the Properties of Asphalt and Bitumen as an Additive. Prog. Rubber Plast. Recycl. Technol..

[14] Xu X., Leng Z., Lan J., Wang W., Yu J., Bai Y., Sreeram A., Hu J. (2021). Sustainable Practice in Pavement Engineering through Value-Added Collective Recycling of Waste Plastic and Waste Tyre Rubber. Engineering.

[15] Wang S., Yuan C., Jiaxi D. (2014). Crumb Tire Rubber and Polyethylene Mutually Stabilized in Asphalt by Screw Extrusion. J. Appl. Polym. Sci..

[16] Fang C., Zhang Y., Yu Q., Zhou X., Guo D., Yu R., Zhang M. (2013). Preparation, Characterization and Hot Storage Stability of Asphalt Modified by Waste Polyethylene Packaging. J. Mater. Sci. Technol..

[17] Yan K., Xu H., You L. (2015). Rheological Properties of Asphalts Modified by Waste Tire Rubber and Reclaimed Low Density Polyethylene. Constr. Build. Mater..

[18] White G., Reid G. (2018). Recycled Waste Plastic for Extending and Modifying Asphalt Binders. Proceedings of the 8th Symposium on Pavement Surface Characteristics (SURF).

[19] Naskar M., Chaki T.K., Reddy K.S. (2010). Effect of Waste Plastic as Modifier on Thermal Stability and Degradation Kinetics of Bitumen/Waste Plastics Blend. Thermochim. Acta.

[20] Ahmad A.F., Razali A.R., Razelan I.S.M. (2017). Utilization of Polyethylene Terephthalate (PET) in Asphalt Pavement: A Review. IOP Conf. Ser. Mater. Sci. Eng..

[21] Silva J.D.A.A.E., Rodrigues J.K.G., de Carvalho M.W., Lucena L.C.D.F.L., Cavalcante E.H. (2018). Mechanical Performance of Asphalt Mixtures Using Polymer-Micronized PET-Modified Binder. Road Mater. Pavement Des..

[22] Dalhat M.A., Al-Abdul Wahhab H.I. (2017). Performance of Recycled Plastic Waste Modified Asphalt Binder in Saudi Arabia. Int. J. Pavement Eng..

[23] Hasan M.R.M., Colbert B., You Z., Jamshidi A., Heiden P.A., Hamzah M.O. (2016). A Simple Treatment of Electronic-Waste Plastics to Produce Asphalt Binder Additives with Improved Properties. Constr. Build. Mater..

[24] Ge D., Yan K., You Z., Xu H. (2016). Modification Mechanism of Asphalt Binder with Waste Tire Rubber and Recycled Polyethylene. Constr. Build. Mater..

[25] Fang C., Zhang M., Yu R., Liu X. (2015). Effect of Preparation Temperature on the Aging Properties of Waste Polyethylene Modified Asphalt. J. Mater. Sci. Technol..

[26] Yu R., Fang C., Liu P., Liu X., Li Y. (2015). Storage Stability and Rheological Properties of Asphalt Modified with Waste Packaging Polyethylene and Organic Montmorillonite. Appl. Clay Sci..

[27] Arabani M., Pedram M. (2016). Laboratory Investigation of Rutting and Fatigue in Glassphalt Containing Waste Plastic Bottles. Constr. Build. Mater..

[28] Yu B., Jiao L., Ni F., Yang J. (2014). Evaluation of Plastic-Rubber Asphalt: Engineering Property and Environmental Concern. Constr. Build. Mater..

[29] Behl A., Sharma G., Kumar G. (2014). A Sustainable Approach: Utilization of Waste PVC in Asphalting of Roads. Constr. Build. Mater..

[30] Okhotnikova E.S., Ganeeva Y.M., Frolov I.N., Yusupova T.N., Firsin A.A. (2018). Plastic Properties and Structure of Bitumen Modified by Recycled Polyethylene. Pet. Sci. Technol..

[31] Aurilio M., Qabur A., Mikhailenko P., Baaj H. (2018). Comparing the Fatigue Performance of HMA Samples with PMA to Their Multiple Stress Creep Recovery and Double Notched Tension Test Properties. Proceedings of the Sixth-Third Annual Conference of the Canadian Technical Asphalt Association (CTAA).

[32] Mikhailenko P., Kadhim H., Baaj H., Tighe S. (2017). Observation of Asphalt Binder Microstructure with ESEM. J. Microsc..

[33] (1996). Superpave Mix Design (SP-2).

[34] (2013). AASHTO T 316 Standard Method of Test for Viscosity Determination of Asphalt Binder Using Rotational Viscometer.

[35] (2019). AASHTO T 315 Standard Method of Test for Determining the Rheological Properties of Asphalt Binder Using a Dynamic Shear Rheometer (DSR).

[36] (2018). AASHTO M 332 Standard Specification for Performance-Graded Asphalt Binder Using Multiple Stress Creep Recovery (MSCR) Test.

[37] Hintz C., Velasquez R., Johnson C., Bahia H. (2011). Modification and Validation of Linear Amplitude Sweep Test for Binder Fatigue Specification. Transp. Res. Rec..

[38] Hintz C., Bahia H. (2013). Simplification of Linear Amplitude Sweep Test and Specification Parameter. Transp. Res. Rec..

[39] Nur N.I., Mounier D., Marc-Stéphane G., Rosli Hainin M., Airey G.D., Di Benedetto H. (2013). Modelling the Rheological Properties of Bituminous Binders Using the 2S2P1D Model. Constr. Build. Mater..

[40] (2020). ASTM D7173 Standard Practice for Determining the Separation Tendency of Polymer from Polymer Modified Asphalt.

[41] Roja K.L., Rehman A., Ouederni M., Krishnamoorthy S.K., Abdala A., Masad E. (2021). Influence of Polymer Structure and Amount on Microstructure and Properties of Polyethylene-Modified Asphalt Binders. Mater. Struct./Mater. Et Constr..

[42] Youtcheff J., Wijayatilleke N., Shenoy A. (2005). Evaluation of the Laboratory Asphalt Stability Test (NCHRP) 9-10.

[43] Behnood A., Gharehveran M.M. (2019). Morphology, Rheology, and Physical Properties of Polymer-Modified Asphalt Binders. Eur. Polym. J..

[44] Deacon J.A., Harvey J.T., Tayebali A., Monismith C.L. (1997). Influence of Binder Loss Modulus on the Fatigue Performance of Asphalt Concrete Pavements. Asphalt Paving Technology 1997.

[45] Chen J.-S., Tsai C.-J. (1999). How Good Are Linear Viscoelastic Properties of Asphalt Binder to Predict Rutting and Fatigue Cracking?. J. Mater. Eng. Perform..

[46] Di Benedetto H., Olard F., Sauzéat C., Delaporte B. (2004). Linear Viscoelastic Behaviour of Bituminous Materials: From Binders to Mixes. Road Mater. Pavement Des..

[47] Zhou F., Mogawer W., Li H., Andriescu A., Copeland A. (2013). Evaluation of Fatigue Tests for Characterizing Asphalt Binders. J. Mater. Civ. Eng..

[48] Subhy A. (2017). Advanced Analytical Techniques in Fatigue and Rutting Related Characterisations of Modified Bitumen: Literature Review. Constr. Build. Mater..

[49] (2020). OPSS MUNI 1101 Municipal Material Specifications.

[50] Sabouri M., Mirzaeian D., Moniri A. (2018). Effectiveness of Linear Amplitude Sweep (LAS) Asphalt Binder Test in Predicting Asphalt Mixtures Fatigue Performance. Constr. Build. Mater..

[51] Qabur A. (2018). Fatigue Characterization of Asphalt Mixes with Polymer Modified Asphalt Cement.

[52] Airey G.D. (2003). Rheological Properties of Styrene Butadiene Styrene Polymer Modified Road Bitumens☆. Fuel.

[53] Ferry J.D. (1980). Viscoelastic Properties of Polymers.

[54] Rodríguez-Alloza A.M., Gallego J., Giuliani F. (2017). Complex Shear Modulus and Phase Angle of Crumb Rubber Modified Binders Containing Organic Warm Mix Asphalt Additives. Mater Struct.

[55] Kakar M.R., Mikhailenko P., Piao Z., Bueno M., Poulikakos L. (2021). Analysis of Waste Polyethylene (PE) and Its by-Products in Asphalt Binder. Constr. Build. Mater..

[56] Yousefi A.A. (2003). Polyethylene Dispersions in Bitumen: The Effects of the Polymer Structural Parameters. J. Appl. Polym. Sci..

[57] McKeen L.W. (2017). Introduction to Plastics and Polymers. Film Properties of Plastics and Elastomers.

